# Sleep symptoms and long-term outcome in adolescents with major depressive disorder: a naturalistic follow-up study

**DOI:** 10.1007/s00787-019-01436-z

**Published:** 2019-11-06

**Authors:** Anna S. Urrila, Olli Kiviruusu, Henna Haravuori, Linnea Karlsson, Satu Viertiö, Jaana Suvisaari, Mauri Marttunen, The Adolescent Depression Study Group

**Affiliations:** 1 Department of Public Health Solutions, Mental Health Unit, National Institute for Health and Welfare, P.O.Box 30, 00271 Helsinki, Finland; 2 Unit of Adolescent Psychiatry, University of Helsinki and Helsinki University Hospital, P.O.Box 83, 00029 HUS Helsinki, Finland; 3 Department of Child Psychiatry, University of Turku and Turku University Hospital, Kiinamyllynkatu 4-8, 20520 Turku, Finland; 4 Department of Clinical Medicine, FinnBrain Birth Cohort Study, University of Turku, Lemminkäisenkatu 3a, Building: Teutori, 20014 Turku, Finland

**Keywords:** Adolescent, Depression, Follow-up, Major depressive disorder, Sleep, Young adult

## Abstract

Sleep abnormalities in major depressive disorder (MDD) have been suggested to represent a vulnerability trait, which might predispose the individual to long-term psychiatric morbidity. In this study, we sought to assess whether the presence of sleep symptoms among adolescents with MDD is associated with poorer long-term outcome in young adulthood during naturalistic follow-up. Adolescent outpatients diagnosed with MDD (*n* = 166; age 13–19 years, 17.5% boys) were followed up during 8 years in naturalistic settings. *N* = 112 adolescents (16.1% boys) completed the 8-year assessment. Sleep symptoms and psychosocial functioning were assessed with structured clinical interviews, and depressive and anxiety symptoms with questionnaires. The severity of sleep symptoms at baseline was not associated with worse outcome at 8 years in terms of any of the outcome measures tested. In particular, the presence of a disturbed sleep–wake rhythm at baseline was associated with a more favourable outcome at 8 years: less depression and anxiety symptoms and higher level of psychosocial functioning. The presence of sleep symptoms in young adulthood was associated with the presence of current depression and anxiety symptoms and poorer psychosocial functioning. The presence of sleep symptoms at follow-up seems to be state-dependent: they are observed in conjunction with other psychiatric symptoms. Contrary to our hypothesis, our results suggest that sleep complaints among adolescents with MDD do not lead to poorer long-term clinical outcome in young adulthood. The link between sleep–wake rhythm disturbance and better long-term outcome needs to be confirmed and examined in detail in further studies, but here we speculate about possible explanations.

## Background

Sleep symptoms are common in depressed adolescents [1, 2]. The presence of sleep problems in depressed adolescents is linked with a more severe clinical picture: a higher severity of depression, occurrence of suicidal thoughts, and worse overall psychosocial functioning [1–3].

Sleep symptoms are also common residual symptoms during recovery from depression [4–8], and their persistent presence during the treatment phase has been associated with depression at 6-month post-treatment follow-up [9]. In our previous study of adolescent outpatients with major depressive disorder (MDD), the presence of sleep symptoms at baseline was not associated with worse 1-year outcome in naturalistic settings: depressed adolescents with sleep symptoms showed steeper clinical improvement than others from their index episode of depression, and the presence of sleep disturbances at follow-up was related to depressive state [10].

It has been theorized, however, that rather than being a state-dependent feature of depression, sleep abnormalities might represent a persistent trait or a vulnerability marker, which might predispose the individual to long-term psychiatric morbidity, including recurrent depression [11–14]. Sleep is designated by the Research Domain Criteria (RDoC) initiative of the National Institute of Mental Health as a transdiagnostic factor which may contribute to the pathophysiology of different psychiatric disorders [15, 16], extending our interest beyond merely depression symptoms to related anxiety symptoms and overall psychosocial functioning. Previous studies have mainly focused on polysomnographic measures of sleep in mixed samples of depressed children and adolescents with a wider age range [13, 14] and in high-risk groups of adolescents [12]. Studies on the extent to which subjective sleep problems are associated with long-term (> 1 year) outcome in depressed adolescents are, to the best of our knowledge, non-existent to date.

In this study, we sought to assess whether the presence of sleep symptoms among adolescents with MDD is associated with poorer long-term (8 years) outcome during naturalistic follow-up. Our hypothesis was that sleep complaints at baseline would be associated with the presence of mood and anxiety symptoms and poorer psychosocial functioning at 8 years and more days spent ill during the 8-year follow-up period. We also assessed the prevalence of sleep symptoms at 8-year follow-up and, further, conducted exploratory analyses on the association of different types of sleep complaints at baseline and 8-year outcome.

## Methods

The present study is part of the Adolescent Depression Study (ADS), which is a naturalistic clinical research study aimed at investigating adolescents’ depressive mood disorders. More detailed descriptions of the recruitment process, participants, assessments, and outcomes have been previously published [17, 18].

### Subjects

At baseline, the Adolescent Depression Study sample comprised of a total of *n* = 218 consecutive outpatients with any depressive mood disorder, of which *n* = 172 suffered from major depressive disorder (MDD) meeting the DSM-IV criteria. Patients with missing/invalid data (*n* = 3), and patients with MDD in full remission already at baseline (*n* = 3) were further excluded, resulting in the inclusion of *n* = 166 adolescent outpatients (age 13–19 years, 17.5% boys) in this study. The exclusion criteria of the ADS study included mental retardation, age under 13 or over 19 years, or insufficient knowledge of the Finnish language. The inclusion criteria and baseline sample characteristics have been described in detail previously [1, 18, 19]. The baseline sample of patients was collected between years 1998 and 2001, and the adolescents were re-evaluated approximately 1 year and approximately 8 years later as young adults [mean time between baseline and 8-year follow-up 8.16 years, standard deviation (SD) 1.01]. The prevalence of sleep complaints, and their association with other psychiatric symptoms at baseline and at 1-year follow-up have been previously examined and published [1, 10], and in this report, we focus on the 8-year follow-up results. *N* = 112 (67.5%; 16.1% boys) of the adolescents completed the 8-year follow-up interview, and analyses presented in this paper are based on their data. Sample characteristics at 8-year follow-up are presented in Table 1.

**Table 1:** Sample characteristics at 8 years for the total sample (*n* = 112)

Sociodemographic characteristics	
Age at 8-year follow-up (mean ± SD)	24.6 ± 1.91
Gender (boy)	16.1
Living conditions	
Alone	26.8
With partner/own children	49.1
With parents	7.1
Other/missing information	17.0
Partnership status	
Married	16.1
Cohabitation	29.5
Divorced/separated	8.9
Unmarried, never cohabited	33.9
Missing information	11.6
Clinical characteristics	
BDI-21 total score (mean ± SD)	7.5 ± 8.5
HDRS total score (mean ± SD)	4.7 ± 5.5
BAI total score (mean ± SD)	7.1 ± 7.9
GAF (mean ± SD)	64.8 ± 14.9
GAF; best during past year (mean ± SD)	66.9 ± 13.5
% Time spent ill during 8 years (mean ± SD)	41.8 ± 28.7
Clinically significant sleep symptoms	18.8
Treatment received between 1 year and 8 years	
Antidepressive medication	58.0
Other psychiatric medication	34.8
Supportive psychotherapy	57.1
Group therapy	13.4
Family counselling	26.8
Inpatient treatment	16.1

Values are expressed as % unless otherwise stated

The items on sleep were excluded from the BDI-21 and HDRS total scores

The study was naturalistic in nature and during the 8-year follow-up period the adolescents thus received “treatment as usual” of clinically defined duration [18]. Between 1-year and 8-year follow-up, of the participants, antidepressant medication was received by *n* = 65 (58.0%; information missing for *n* = 1), other psychiatric medication by *n* = 39 (34.8%; information missing for *n* = 2), supportive psychotherapy by *n* = 64 (57.1%), group therapy by *n* = 15 (13.4%), family counselling by *n* = 30 (26.8%, information missing for *n* = 1), and *n* = 18 (16.1%) received inpatient treatment at least once (Table 1).

The subjects gave written informed consent to participate and the study protocol was approved by the ethics committees of Helsinki University Central Hospital and Peijas Medical Health Care District.

### Assessment of psychiatric symptoms and level of functioning

During the baseline and follow-up interviews, the participants were assessed with structured diagnostic interviews (K-SADS-PL at baseline and SCID-I at 8-year follow-up) [20, 21]. The diagnoses were confirmed in a diagnostic meeting and inter-rater reliability was good for mood disorders based on randomly selected videotaped interviews [weighted Ƙ for a three-category variable (1 = MDD, 2 = other mood disorder, 3 = no mood disorder) 0.87; 95% confidence interval (CI) 0.81, 0.93] [19, 22]. Time spent ill during the follow-up period (% of time) was assessed with the clinical interview.

Overall psychosocial functioning was assessed during baseline and follow-up with the global assessment of functioning scale (GAF; numeric range of 0–100) as part of the DSM-IV axial diagnostic procedure according to DSM-IV guidelines [23]. At 8 years, also best overall psychosocial functioning during the past 1 year was scored and analysed.

Depression symptom severity was assessed with the 21-item Beck Depression Inventory (BDI-21) and the Hamilton Depression Rating Scale (HDRS). BDI-21 is a standardized self-report questionnaire which has been well studied in both adolescents and adults [24–26], while the HDRS is a clinician-administered depression rating scale [27]. In the analyses presented in this paper, the BDI-21 and HDRS items on sleep were excluded because we were interested in the relationship between sleep and other depression symptoms, and consequently the maximum score for BDI-21 in our analyses was 60 points and for HDRS 46 points.

Anxiety symptoms were assessed with the 21-item Beck Anxiety Inventory (BAI), a self-report measure of anxiety symptoms with a maximum score of 63 points [28].

### Assessment of sleep symptoms

At baseline, sleep complaints were assessed with the K-SADS-PL attachment for assessment of affective disorders, which includes six items about the following sleep symptoms: initial insomnia, middle insomnia, terminal insomnia, sleep–wake rhythm disturbance, non-restorative sleep, and hypersomnia. The interviewer rates each symptom as non-existent, sub-threshold, or clinically significant according to standard criteria, explained in detail previously [1].

For assessment of sleep symptom severity at baseline, a continuous measure (sleep complaint severity score) was formed based on the total sum of all six K-SADS-PL sleep item scores at baseline (1–3 points per item, minimum 6 points, maximum 18 points; missing answers (*n* = 4 in total) were substituted with the average points of all items of the respective participant’s answers). The assessment of sleep complaint severity was identical to that used in our previous publication [10]. The participants were then divided into three sleep subgroups based on the amount sleep complaints at baseline; subgroups A (6–9 points; *n* = 33; no/mild sleep complaints), B (10–13 points; *n* = 61; moderate sleep complaints), and C (14–18 points; *n* = 18; severe sleep complaints). As an alternative approach, we further compared the adolescents with multiple sleep disturbances (*n* = 32) at baseline with those with no or only one complaint of sleep disturbance (*n* = 76), which was similarly an approach used in our previous publication [1].

As the K-SADS-PL was not administered in young adulthood, an approximate of the presence/absence of clinically significant sleep symptoms at 8-year follow-up was obtained with the SCID-I interview (one item on sleep disturbances; hypersomnia/insomnia nearly every day for 2 weeks; rated as non-existent/sub-threshold/clinically significant symptoms). All items related to MDD (including the item on sleep) were asked from all participants regardless of the amount of reported core symptoms of MDD.

### Statistical analyses

Statistical analyses were performed with the IBM SPSS Statistics Version 25. To assess cross-sectional differences between sleep subgroups Chi square tests and independent samples Mann–Whitney *U* tests (non-normal distribution of the data) were used. To assess the associations between baseline sleep complaints and 8-year outcomes, a series of linear regression analyses were performed. In regression analyses, logarithmic transformations were performed for all parameters with a skewed distribution before entering the parameters in the model. First, the crude univariate associations were analysed. Second, the models were adjusted for gender and age at baseline. Third, the models were adjusted for gender, age, and value of respective outcome variable at baseline when applicable. Finally, the model was adjusted for gender, age at baseline, and baseline values of BDI-21, BAI, and GAF, except for outcome HDRS at 8 years, where the model was adjusted for gender, age at baseline, and baseline values of HDRS (instead of BDI-21), BAI, and GAF. For all analyses, a *p* value of < 0.05 was considered statistically significant.

## Results

### Sleep symptoms at 8 years and other psychiatric symptoms

At 8 years, 18.8% (*n* = 21) of the participants suffered from clinically significant sleep symptoms, while 20.5% (*n* = 23) had sub-threshold sleep symptoms, and the rest (*n* = 68; 60.7%) did not report any sleep symptoms. As compared with participants without clinically significant sleep symptoms at 8 years, those suffering from clinically significant sleep symptoms at 8 years had higher depression symptom severity (BDI-21 and HDRS total score), worse current and past 1 year psychosocial functioning, and higher anxiety symptom severity (BAI) (independent samples Mann–Whitney *U* tests *p* < 0.001), while no significant difference was observed in time spent ill during the total follow-up period (independent samples Mann–Whitney *U* test n.s.).

### Baseline sleep complaints and 8-year outcome

**Fig. 1:**
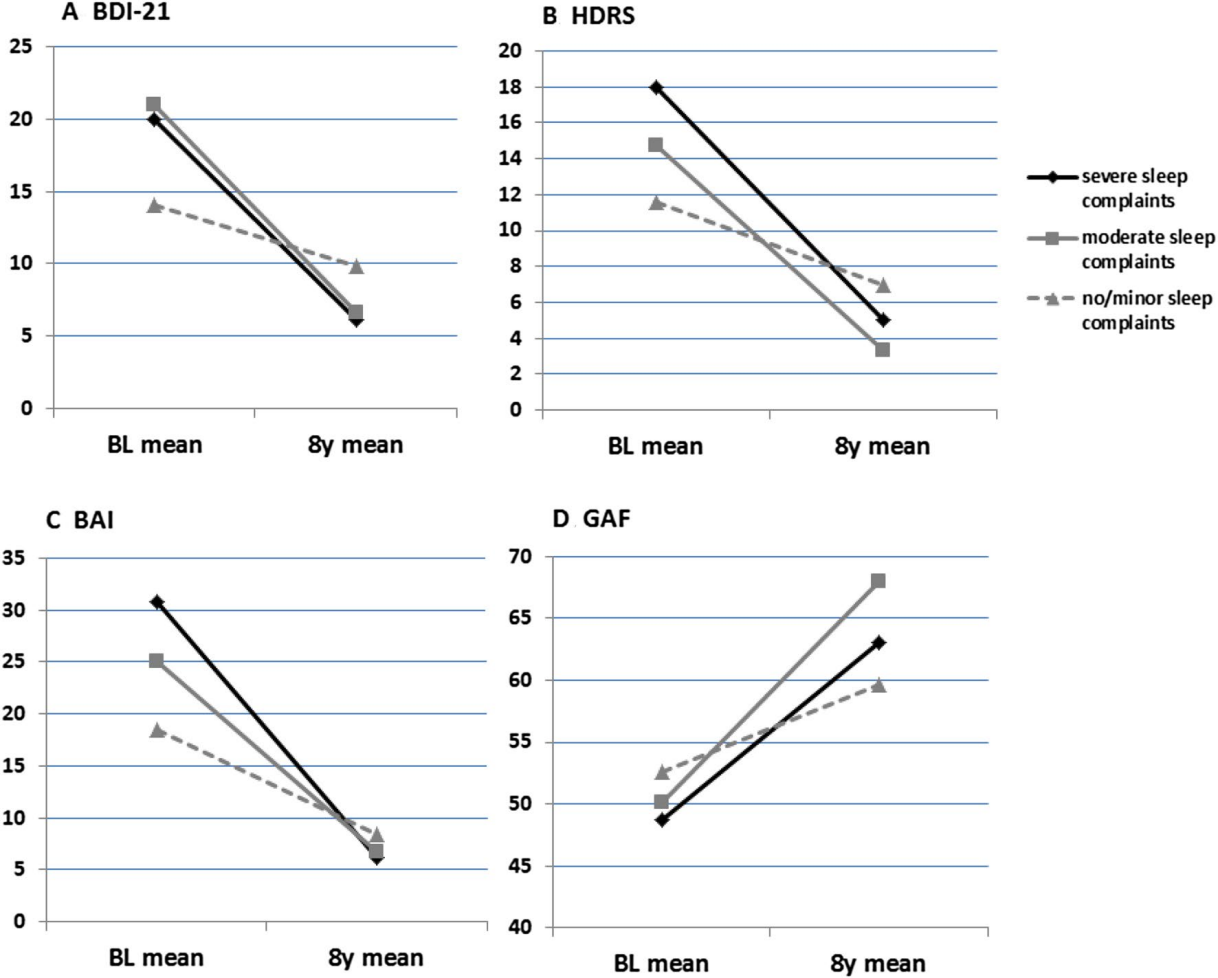
Depression and anxiety symptom severity and psychosocial functioning during follow-up according to baseline sleep complaint severity subgroup. Black lines represent patients with severe sleep complaints at baseline (sleep complaint severity score 14–18p; *n* = 18), grey lines patients with moderate sleep symptoms at baseline (10–13p; *n* = 61), and grey dotted lines patients with no or mild sleep complaints at baseline (6–9p; *n* = 33). *BL* baseline. **a** BDI-21 and **b** HDRS subgroup mean total scores without sleep items are presented

The results of the regression analyses are shown in Tables 2 and 3. Baseline sleep complaint severity subgroup was associated with BDI-21 and HDRS total score at 8 years as well as GAF at 8 years consistently across all regression models tested (Table 2; Fig. 1). Baseline sleep complaint severity subgroup was not associated with BAI at 8 years and best GAF during the past 1 year at 8 years in the crude univariate model, but when the analyses were controlled for baseline values of BAI and best GAF during past one year, respectively, both associations turned statistically significant (adjusted model 2), and the association remained significant also in model 3 when other baseline covariates were controlled for (Table 2). No association between sleep complaint severity subgroup and time spent ill during the follow-up period was observed in any of the models. These associations were found when comparing subgroups A (no/minor sleep complaints) and B (moderate sleep complaints) so that belonging to subgroup B was consistently associated with better outcomes than belonging to subgroup A. Belonging to subgroup C (severe sleep complaints) vs. subgroup A was not associated with any of the outcomes tested.

**Table 2:** Baseline sleep complaint severity and clinical outcome at 8 years; regression analyses

Outcome at 8 years	Crude model		Adjusted model 1		Adjusted model 2		Adjusted model 3	
	*B* (SE)	*p*	*B* (SE)	*p*	*B* (SE)	*p*	*B* (SE)	*p*
BDI-21 total score at 8 years								
Subgroup B	− 0.23 (0.10)	0.03*	− 0.29 (0.12)	0.02*	− 0.33 (0.12)	0.007*	− 0.35 (0.13)	0.007*
Subgroup C	− 0.22 (0.14)	0.14	− 0.29 (0.16)	0.07	− 0.30 (0.15)	0.06	− 0.23 (0.18)	0.20
HDRS total score at 8 years								
Subgroup B	− 0.27 (0.09)	0.004*	− 0.31 (0.11)	0.004*	− 0.34 (0.10)	0.001*	− 0.36 (0.11)	0.002*
Subgroup C	− 0.11 (0.13)	0.36	− 0.17 (0.13)	0.21	− 0.26 (0.13)	0.06	− 0.25 (0.16)	0.11
BAI total score at 8 years								
Subgroup B	− 0.09 (0.10)	0.37	− 0.12 (0.11)	0.29	− 0.24 (0.12)	0.04*	− 0.24 (0.12)	0.04*
Subgroup C	− 0.07 (0.14)	0.59	− 0.11 (0.15)	0.46	− 0.20 (0.16)	0.21	− 0.19 (0.16)	0.25
GAF at 8 years								
Subgroup B	0.07 (0.03)	0.01*	0.07 (0.03)	0.02*	0.08 (0.03)	0.01*	0.09 (0.03)	0.005*
Subgroup C	0.03 (0.04)	0.44	0.03 (0.04)	0.43	0.04 (0.04)	0.31	0.03 (0.05)	0.50
GAF at 8 years; best during past year								
Subgroup B	0.04 (0.02)	0.08	0.05 (0.03)	0.06	0.06 (0.03)	0.02*	0.07 (0.03)	0.01*
Subgroup C	0.01 (0.03)	0.85	0.01 (0.03)	0.74	0.03 (0.03)	0.40	0.03 (0.04)	0.44
% Time spent ill during 8 years								
Subgroup B	− 0.06 (0.07)	0.43	− 0.08 (0.08)	0.37	NA	NA	− 0.13 (0.09)	0.16
Subgroup C	− 0.09 (0.10)	0.41	− 0.07 (0.11)	0.50	NA	NA	− 0.07 (0.13)	0.57

Subgroup A = patients with no/minor sleep complaints (sleep complaint severity score 6–9p; *n* = 33) was chosen as the reference group, subgroup B = patients with moderate sleep disturbances at baseline (10–13p; *n* = 61), subgroup C = patients with severe sleep disturbances at baseline (14–18p; *n* = 18)

Adjusted model 1 has been adjusted for gender and age at baseline

Adjusted model 2 has been adjusted for gender, age at baseline, and value of respective outcome variable at baseline

Adjusted model 3 has been adjusted for gender, age at baseline, and baseline values of BDI-21, BAI, and GAF, except for outcome HDRS at 8 years, where the model was adjusted for gender, age at baseline, and baseline values of HDRS, BAI, and GAF

*SE* standard error, *NA* not applicable

*p < 0.05

**Table 3:** Baseline multiple sleep disturbances and clinical outcome at 8 years; regression analyses

Outcome at 8 years	Crude univariate model		Adjusted model 1		Adjusted model 2		Adjusted model 3	
	*B* (SE)	*p*	*B* (SE)	*p*	*B* (SE)	*p*	*B* (SE)	*p*
BDI-21 total score at 8 years	− 0.23 (0.10)	0.03*	− 0.25 (0.11)	0.03*	− 0.25 (0.11)	0.03*	− 0.27 (0.12)	0.03*
HDRS total score at 8 years	− 0.10 (0.09)	0.27	− 0.13 (0.10)	0.22	− 0.14 (0.10)	0.15	− 0.15 (0.11)	0.17
BAI total score at 8 years	− 0.25 (0.09)	0.008*	− 0.24 (0.10)	0.02*	− 0.24 (0.10)	0.02*	− 0.24 (0.11)	0.03*
GAF at 8 years	0.03 (0.03)	0.22	0.02 (0.03)	0.41	0.03 (0.03)	0.25	0.04 (0.03)	0.27
GAF at 8 years; best during past year	0.02 (0.02)	0.33	0.02 (0.02)	0.54	0.03 (0.02)	0.22	0.03 (0.03)	0.21
% Time spent ill during 8 years	− 0.05 (0.07)	0.51	− 0.06 (0.08)	0.46	NA	NA	− 0.08 (0.09)	0.36

Subgroup of patients with no/only minor sleep disturbances at baseline (*n* = 80) was chosen as the reference group as compared to patients with multiple sleep disturbances at baseline (*n* = 32)

Adjusted model 1 has been adjusted for gender and age at baseline

Adjusted model 2 has been adjusted for gender, age at baseline, and value of respective outcome variable at baseline

Adjusted model 3 has been adjusted for gender, age at baseline, and baseline values of BDI-21, BAI, and GAF, except for outcome HDRS at 8 years, where the model was adjusted for gender, age at baseline, and baseline values of HDRS, BAI, and GAF

*SE* standard error, *NA* not applicable

*p < 0.05

In the regression analyses using the alternative subgroups, the presence of multiple sleep disturbances at baseline was associated with BDI-21 at 8 years and BAI at 8 years consistently across all tested models (Table 3). No other statistically significant associations between the presence of multiple sleep disturbances at baseline and 8-year outcomes were observed in any of the models.

Drop-out rates during the 8-year follow-up period did not differ between the studied sleep subgroups (Chi square tests n.s.).

### Type of sleep complaint and 8-year outcome

As we got unexpected results when comparing 8-year outcomes of sleep subgroups (adolescents with moderate sleep complaints and adolescents with multiple sleep disturbances at baseline showing better outcome as young adults), we decided to perform additional exploratory analyses to assess whether a specific type of sleep complaint (insomnia, hypersomnia, or sleep–wake rhythm disturbance) at baseline would be associated with a particularly favourable long-term outcome. In these analyses, adolescents with clinically relevant insomnia (*n* = 61), hypersomnia (*n* = 24), and sleep–wake rhythm disturbance (*n* = 28) at baseline were compared with the group of patients who did not present with the respective sleep disturbance at baseline.

These analyses showed that the adolescents with clinically relevant sleep–wake rhythm disturbance at baseline (sleeping at a different time of the day than usual more than once a week) differed significantly from those who did not present with sleep–wake rhythm disturbance at baseline in terms of the following 8-year outcomes: BDI-21 total score (independent samples Mann–Whitney *U* test *p* = 0.008), HDRS total score (*p* = 0.007), GAF (*p* = 0.002), best GAF during past year (*p* = 0.005), and BAI total score (*p* = 0.016), adolescents with sleep–wake rhythm disturbance at baseline having consistently better outcomes at 8 years than adolescents without a sleep–wake rhythm disturbance. Adolescents with insomnia or hypersomnia at baseline did not differ significantly from adolescents without these disturbances in terms of 8-year outcome (independent samples Mann–Whitney *U* tests n.s.). In all regression analysis models tested, all tested 8-year outcomes were associated with the presence of a sleep–wake rhythm disturbance at baseline, with the exception of time spent ill during the 8-year follow-up period, which was not associated with the presence of sleep–wake rhythm disturbance at baseline in any of the models (Table 4).

**Table 4:** Baseline sleep–wake rhythm disturbance and outcome at 8 years

Outcome at 8 years	Crude univariate model		Adjusted model 1		Adjusted model 2		Adjusted model 3	
	*B* (SE)	*p*	*B* (SE)	*p*	*B* (SE)	*p*	*B* (SE)	*p*
BDI-21 total score at 8 years	− 0.28 (0.10)	0.008*	− 0.30 (0.11)	0.009*	− 0.29 (0.11)	0.01*	− 0.31 (0.12)	0.01*
HDRS total score at 8 years	− 0.24 (0.10)	0.02*	− 0.27 (0.10)	0.008*	− 0.29 (0.10)	0.005*	− 0.27 (0.11)	0.01*
BAI total score at 8 years	− 0.24 (0.10)	0.01*	− 0.23 (0.10)	0.03*	− 0.24 (0.11)	0.03*	− 0.25 (0.11)	0.02*
GAF at 8 years	0.08 (0.03)	0.004*	0.08 (0.03)	0.007*	0.09 (0.03)	0.003*	0.08 (0.03)	0.01*
GAF at 8 years; best during past 1 year	0.07 (0.02)	0.004*	0.07 (0.02)	0.007*	0.08 (0.02)	0.001*	0.08 (0.03)	0.004*
% Time spent ill during 8 years	− 0.04 (0.08)	0.64	− 0.03 (0.08)	0.70	NA	NA	− 0.04 (0.09)	0.64

The subgroup of patients without sleep–wake rhythm disturbance at baseline (*n* = 84) was chosen as the reference group as compared to patients with sleep–wake rhythm disturbance at baseline (*n* = 32)

Adjusted model 1 has been adjusted for gender and age at baseline

Adjusted model 2 has been adjusted for gender, age at baseline, and value of respective outcome variable at baseline

Adjusted model 3 has been adjusted for gender, age at baseline, and baseline values of BDI-21, BAI, and GAF, except for outcome HDRS at 8 years, where the model was adjusted for gender, age at baseline, and baseline values of HDRS, BAI, and GAF

*SE* standard error, *NA* not applicable

*p < 0.05

As supplementary analyses, we first compared background information of adolescents with sleep–wake rhythm disturbance at baseline and the rest of the adolescents. These two subgroups did not differ in terms of gender distribution, age at baseline, parental socio-economic status, parental divorce rate, perceived psychosocial support, recurrence/length of depression at baseline, level of depression/anxiety at baseline, psychosocial functioning at baseline, treatments received during the 8-year follow-up, and drop-out rate during 8-year follow-up (independent samples Mann–Whitney *U* tests and *χ*^2^ tests n.s.). Second, we wanted to see if normalization of the sleep–wake rhythm during the first 1 year of the follow-up would be associated with 8-year outcome. In these analyses, we included only those participants whose data were available in all three time points (baseline, 1 years, 8 years; *n* = 97). The group of adolescents whose initial sleep–wake rhythm disturbance remitted during the 1-year follow-up (*n* = 18) had a more favourable 8-year outcome than the rest of the adolescents (*n* = 79) (Table 5). The number of adolescents suffering from a persistent sleep–wake rhythm disturbance from baseline to 1 year was unfortunately too small (*n* = 4) to be included in the statistical models.

**Table 5:** Normalization of disturbed sleep–wake rhythm during 1 year and outcome at 8 years

Measure at 8 years	Crude univariate model		Adjusted model 1		Adjusted model 2		Adjusted model 3	
	*B* (SE)	*p*	*B* (SE)	*p*	*B* (SE)	*p*	*B* (SE)	*p*
BDI-21 total score at 8 years	− 0.29 (0.12)	0.02*	− 0.30 (0.13)	0.02*	− 0.30 (0.13)	0.03*	− 0.30 (0.14)	0.04*
HDRS total score at 8 years	− 0.23 (0.11)	0.04*	− 0.25 (0.12)	0.04*	− 0.27 (0.12)	0.03*	− 0.27 (0.13)	0.04*
BAI total score at 8 years	− 0.25 (0.12)	0.03*	− 0.25 (0.12)	0.04*	− 0.26 (0.13)	0.04*	− 0.27 (0.13)	0.04*
GAF at 8 years	0.07 (0.03)	0.03*	0.07 (0.04)	0.04*	0.07 (0.04)	0.05*	0.07 (0.04)	0.10
GAF at 8 years; best during past y	0.07 (0.03)	0.02*	0.07 (0.03)	0.02*	0.07 (0.03)	0.02*	0.06 (0.03)	0.05*
% Time spent ill during 8 years	− 0.08 (0.09)	0.42	− 0.06 (0.10)	0.54	NA	NA	− 0.03 (0.10)	0.74

As supplementary analyses, the depressed adolescents with sleep–wake rhythm at baseline who did not show this symptom anymore at 1-year follow-up (*n* = 18) were compared with the rest of the adolescents (*n* = 79; reference category)

Adjusted model 1 has been adjusted for gender and age at baseline

Adjusted model 2 has been adjusted for gender, age at baseline, and value of respective outcome variable at baseline

Adjusted model 3 has been adjusted for gender, age at baseline, and baseline values of BDI-21, BAI, and GAF, except for outcome HDRS at 8 years, where the model was adjusted for gender, age at baseline, and baseline values of HDRS, BAI, and GAF

*SE* standard error, *NA* not applicable

*p < 0.05

## Discussion

The main and unexpected finding of this naturalistic clinical long-term (8 years) follow-up study was that the presence of sleep symptoms among depressed adolescents was not associated with poorer long-term clinical outcome. On the contrary, baseline sleep–wake rhythm disturbance was associated with a particularly good clinical outcome at 8 years: less depression and anxiety symptoms and higher level of psychosocial functioning.

In addition, the overall prevalence of sleep symptoms among the depressed adolescents in young adulthood (18.8%) was expectedly lower than at baseline (prevalence of clinically significant sleep symptoms 83%) [1] and 1-year follow-up (50%) [10]. As the presence of sleep symptoms in young adulthood was associated with the presence of current depression and anxiety symptoms and poorer psychosocial functioning, but not with time spent ill during the total study period, the findings of this study support the state-dependent association of sleep symptoms with other current psychiatric morbidity [9].

Our main finding is in line with our previous report in the same sample of participants at 1-year follow-up [10], but contradicts with previous findings pointing towards sleep abnormalities acting as predisposing factors to psychiatric morbidity and poor social functioning [11–14, 16, 29]. This discrepancy might be explained mainly by the considerably longer follow-up time in our current study, as well as the differing methods for assessing sleep abnormalities (objective sleep EEG vs. subjective reports). Polysomnographic studies in MDD point overall towards alterations in sleep continuity, sleep depth, and REM sleep pressure [30, 31], the findings being less consistent and less severe among children and adolescents than among adults [30–32]. In turn, subjective sleep complaints among depressed adolescents include insomnia or hypersomnia, experiences of insufficient sleep quality, and daytime sleepiness [29, 31]. Based on the current study, we cannot rule out the possible presence/absence of, e.g., REM sleep changes, alterations of slow wave sleep (SWS), or sleep EEG coherence abnormalities in our sample. It is possible, that the EEG abnormalities might present a trait-like phenomenon, while the subjective sleep complaints would be more state-dependent. Despite the partial discrepancy between the subjective and objective findings, subjective sleep measures provide a clinically more useful tool to address sleep problems experienced by the patients [33].

As most of our outcome measures at 8 years were cross-sectional, the current study cannot answer more precise questions on the relationship between sleep symptoms and trajectories of depression during the 8-year follow-up period. As the sleep subgroups did not differ in terms of total time spent ill during the 8-year follow-up period, it seems likely that the adolescents with disturbed sleep at baseline were not constantly feeling better than the rest of the participants. The adolescents with disturbed sleep–wake rhythm at baseline showed, however, higher level of psychosocial functioning during the past 1 year before the 8 years assessment, meaning that the association between sleep disturbances at baseline and outcome at 8 years was not purely limited to the 8-year time point.

The especially good 8-year outcome of depressed adolescents with sleep–wake rhythm disturbance was unexpected. In theory, this finding might be explained by several factors unrelated to the nature of the sleep disturbance itself. No differences were, however, observed between adolescents with sleep–wake rhythm disturbance and the rest of the adolescents in background profiles, baseline psychiatric symptoms, and treatment received during 8 years.

If we assume that the sleep–wake rhythm disturbance itself would have contributed to the onset of depression in adolescence, it is possible that the physiological stabilization and advance of circadian rhythms when getting older [34] could have benefitted the adolescents and led over time to alleviation of other psychiatric symptoms as well. Unfortunately, no information on 8-year sleep–wake rhythm was available in our study. However, supporting this idea, in our sample of depressed adolescents, normalization of the sleep–wake rhythm during the first year of the follow-up was associated with a favourable 8-year outcome. It must also be noted that, in contrast to other sleep disturbances, sleep EEG structure itself is usually well maintained in sleep–wake rhythm disturbances [35, 36], potentially making the group of adolescents with a disturbed sleep–wake rhythm a unique subgroup among the depressed adolescents. Further, since manipulations of the sleep–wake rhythm can be used as treatments for affective disorders [37], we can speculate that the deviations of the sleep–wake rhythm at baseline could have represented actually an (successful) attempt of the depressed adolescents to cope with their affective symptoms. As the baseline sample was collected in years 1998–2001 before the dramatic increase in electronic media use among adolescents in Finland, the cause for the adolescents’ sleep–wake rhythm disturbance was most likely unrelated to excessive electronic media use. Ultimately, the current findings highlight the complex link between sleep, circadian rhythms, and the regulation of mood.

The major strengths of this study include the clinically well-characterized sample of adolescents with long-term follow-up in naturalistic settings, the homogeneous age range and the use of standardized psychiatric evaluation instruments. Limitations include most notably the lack of objective measures of sleep, and lack of sleep-specific questionnaires and sleep logs (no information available on, e.g., habitual sleep length), as well as the lack of more detailed information on sleep symptoms at 8-year follow-up. Further limitations are the use of the GAF scale to assess psychosocial functioning, which does not allow disentangling the aspects of psychopathology and social functioning, and the lack of detailed information of medication use (e.g., specific type of antidepressant, duration of medication). Finally, the over-representation of girls in our sample may limit the generalizability of the findings to depressed adolescent boys.

## Conclusions

Baseline sleep disturbances did not have an unfavourable effect on clinical outcome in an 8-year naturalistic clinical follow-up of depressed adolescents, suggesting that subjective sleep disturbances at baseline do not lead to poorer clinical outcome in young adulthood. In particular, sleep–wake rhythm disturbance was associated with a good outcome within the group of depressed adolescents. To confirm these findings and to clarify the link between sleep and depression in adolescents in further detail, future studies with larger sample sizes and objective measures of sleep and circadian rhythm should be conducted. In particular, the link between different types of sleep disturbances and clinical outcome warrants attention.
